# The Effects of Ondansetron on the Analgesic Action of Intravenous Acetaminophen after Tonsillectomy in Children: A Triple-Blind Randomized Controlled Trial

**DOI:** 10.1155/2021/6611740

**Published:** 2021-04-23

**Authors:** Mahshid Nikooseresht, Maryam Nasrolahi, Pouran Hajian, Abbas Moradi

**Affiliations:** ^1^Department of Anesthesiology, Hamadan University of Medical Sciences, Hamadan, Iran; ^2^Student Research Committee, Hamadan University of Medical Sciences, Hamadan, Iran; ^3^Department of Community Medicine, Hamadan University of Medical Sciences, Hamadan, Iran

## Abstract

**Introduction:**

Severe pain, nausea, and vomiting after tonsillectomy surgery are among the issues that not only affect patient satisfaction but also may result in complications and delay patient discharge. This study was conducted to assess the effect of intravenous administration of ondansetron on the analgesic action of intravenous acetaminophen after tonsillectomy in children. *Materials and methods*. This randomized controlled trial was conducted on 53 children between the age of 3 and 12 years old who were referred to Besat Hospital Hamadan, Iran, for tonsillectomy. Patients were randomly assigned to two groups. The intervention group (I) received intravenous acetaminophen plus 0.1 mg/kg ondansetron intravenously while the control group (C) received intravenous acetaminophen plus 2 ml of normal saline intravenously. Postoperative pain severity was assessed using the Children's Hospital Eastern Ontario Pain Scale (CHEOPS). Frequency of nausea, vomiting, and need for analgesic was assessed and recorded four times, at recovery unit, after 6, 12, and 24 hours postsurgery. Data analysis was performed at 95% confidence level using the statistical package for social sciences (SPSS) software version 21.

**Results:**

The number of patients in groups I and C was 27 and 26 patients, respectively. Mean pain score in I and C groups was 4.48 and 2.88 at recovery unit, 2.74 and 2.04 after 6 hours, 1.67 and 0.81 after 12 hours, and 0.67 and 0.20 after 24 hours postsurgery, respectively. Frequency of nausea at recovery unit was 23.1% in I group and 0.0% in group C (*p* = 0.010) while the mean number of analgesic requirements in I and C groups was 1.07 and 0.56 times, respectively (*p* = 0.027).

**Conclusions:**

Intravenous administration of 0.1 mg/kg ondansetron reduces the analgesic action of intravenous acetaminophen after tonsillectomy in 3 to 12-year-old children.

## 1. Introduction

Infectious diseases, tonsillitis, pharyngitis, and adenoid inflammation are among the most important ailments in pediatrics that require care in this period of life. Chronic inflammation of tonsils, which is called tonsillitis, is a common condition that may or may not be accompanied by adenoid enlargement. Chronic tonsillitis can result in recurrent sore throat, fever, dysphagia, or fatigue [1]. Nearly 4% of children with chronic tonsillitis experience obstructive sleep apnea, which is characterized by the collapse of the upper respiratory tract during the sleep [2, 3]. Another common and important complaint of children with chronic tonsillitis is pain. Pain may result in long-term suffering of the child and may result in weakness due to refusal to eat or drink. Therefore, tonsillectomy is suggested for children with recurrent episodes of inflammation and infection in tonsils for consecutive years [4]. Tonsillectomy is one of the most frequent pediatric surgeries in the United States, and 530000 tonsillectomy operations are performed annually in the United States [5, 6].

Pain management is one of the main challenges in pediatric. Postoperational nausea and vomiting are among the key elements that affect the quality of postoperational recovery. Despite the use of various pain management strategies by the anesthesiologists, postoperative pain is still one of the major complaints in children undergoing tonsillectomy. The consequences of postoperative pain in children, especially in the preschool period, are more than adults. Presence of pain, nausea, and vomiting not only may result in complications in recovery but also may delay the discharge of the child. Therefore, a wide range of analgesics have been suggested for pain management in this age group [7, 8].

Monotherapy with opioids is a common method for postoperative pain management. The side effects of opioid administration, including nausea, vomiting, and ileus, can occur even at low doses of opioid and may result in respiratory depression and increased risk for aspiration. Therefore, medications with fewer side effects are prioritized. Many studies have assessed the efficacy of different medications in postoperative pain management. The study medications included nonsteroid anti-inflammatory drugs (NSAIDs), acetaminophen, opioids, ketamine, dextromethorphan, and local intralesional analgesics. Other studies proposed analgesic effects for diclofenac [9], tramadol infiltration, other opioids, fentanyl, acetaminophen, and ketamine in the prevention of posttonsillectomy pain [10].

Acetaminophen is one of the effective analgesics in postoperative pain management. Safety and tolerance profile of acetaminophen is among its superiorities over other analgesic medications. Furthermore, acetaminophen is approved by the Food and Drug Administration (FDA). Acetaminophen produces its effects by influencing on opioid receptors but dies not possessing the side effects of opioids and therefore results in less drowsiness and respiratory depression. Ileus and constipation were compared to opioids. Furthermore, in comparison to NSAIDs, acetaminophen produces less renal and coagulopathic complications after surgery. Intravenous administration of acetaminophen can result in a fast elevation in acetaminophen plasma concentration, and the peak analgesic effect is achieved during one hour and lasts for about 4-6 hours. Acetaminophen affects on serotonergic pathways and inhibits pain signals to spinal pain receptors. Other medications that affect serotonergic pathways, including ondansetron, may also be administered besides acetaminophen for pain management [11].

Ondansetron is a 5-hydroxytryptamine 3 (5-HT3) antagonist and primarily affects chemoreceptors in excitatory neurons in the medulla oblongata. Ondansetron modulates pain signals through affecting the 5-HT3 receptors and is an antagonist for acetaminophen, as both medications have opposite effects on serotonergic pathways [12]. Although laboratory assessments have hypothesized low probability of antagonistic effects for coadministration of acetaminophen and ondansetron, this issue has not been assessed in clinical settings. As both acetaminophen and ondansetron are widely used postoperatively and due to the possibility of their antagonist effects of 5-HT3 receptors, this study was conducted to assess the effect of acetaminophen ondansetron coadministration on posttonsillectomy pain.

## 2. Materials and Methods

### 2.1. Study Population

This triple-blind randomized controlled trial was conducted in Besat Hospital, Hamadan, Iran, during 2018-2019. The sampling frame included all children between the age of 3 and 12 years old who were candidate for tonsillectomy under general anesthesia. The sampling method in this study was random sampling, and samples were selected from patients in American Society of Anesthesiologists (ASA) classes I and II.

Sample size was calculated based on the findings of the study by Ramirez et al. [6], which reported the percentage of opioid administration in patients who received acetaminophen and ondansetron as 57.1% and in patients who received acetaminophen alone as 20.6%, considering power = 80% and alpha error = 0.05 using the following inequality equation two sided; the sample size was calculated as 23 patients in each group.

(1) $$\begin{array}{c} n=\frac{{\left(Z_{1-\alpha /2}+Z_{1-\beta }\right)}^{2}.\left[P_{1}\left(1-P_{1}\right)+P_{2}\left(1-P_{2}\right)\right]}{{\left(P_{1}-P_{2}\right)}^{2}}. \end{array}$$

In order to compensate falling of data, 27 patients were enrolled in each study group and finally 26 patients in the control group and 27 in the intervention group completed the study.

### 2.2. Inclusion Criteria

The inclusion criteria are as follows: being candidate for elective adenotonsillectomy, willingness of the parents or guardians, patient cooperation after surgery, and consciousness of the patient.

### 2.3. Exclusion Criteria

The exclusion criteria are as follows: being unable to describe pain, aggravated conscious state, use of analgesics 24 hours prior to surgery, allergy to acetaminophen or ondansetron, history for liver disease, and psychological or neurological disorders.

### 2.4. Ethical Considerations

This study was approved by the Ethical Committee of the Hamadan University of Medical Sciences (IRCT.UMSHA.REC1398.433) and was registered in the Iranian Registry of Clinical Trials (IRCT20160508027793N1). A written informed consent was obtained from patients' parents or guardians after providing them with necessary information regarding the research. The research protocol was designed based on the Declaration of Helsinki, and patient information was recorded anonymously for confidentiality.

### 2.5. Data Collection

In this triple-blind randomized controlled trial, a questionnaire comprising of demographic data, including age, weight, and gender, was filled for each patient. Then, patients were randomly assigned to intervention and control groups. All patients, regardless of the treatment group, received similar premedication including midazolam 1-2 mg/kg plus atropine 0.02 mg/kg 5 minutes prior to the induction of anesthesia. Anesthesia was induced using fentanyl 1.5 *μ*g/kg, sodium thiopental 5 mg/kg, and atracurium 0.5 mg/kg. Patients were then intubated. Then, dexamethasone was administered at 0.015 mg/kg, and anesthesia was maintained using 50% NO_2_/O_2_ and isoflurane gas at the minimum alveolar concentrations (MAC) of 1%. Intravenous acetaminophen was administered at 15 mg/kg during 15 minutes in both groups and 15 minutes prior to the termination of surgery. The intervention group received 0.1 mg/kg ondansetron (at the volume of 2 ml) simultaneously but the control group received 2 ml of normal saline (equal volume). Both groups received rectal acetaminophen at 15 mg/kg every 6 hours for 24 hours. Similar surgical technique, sharp dissection with snake, was performed for all patients, and electrocautery was used for hemostasis. Neuromuscular block was reversed by the administration of neostigmine (0.045 mg/kg) and atropine (0.02 mg/kg) after surgery. The patient was extubated and was sent to the recovery unit after achieving regular and sufficient breathing. Severity of pain was assessed in the recovery unit using the Children's Hospital Eastern Ontario Pain Scale (CHEOPS). This scale evaluates pain severity based on crying, facial expression, verbal responses, torso, wound evaluation, and leg position. Patients are scored based on a scoring table ranging from a scale of 0 to 100. Data was collected by a research assistant who was blinded regarding the group allocation. Furthermore, symptoms including nausea, vomiting, and agitation as well as airway problems, spasm, and hemodynamic instability were also recorded in a checklist during the patient stay in recovery unit. Later on, pain severity, nausea, and vomiting were recorded in a similar method after 6, 12, and 24 hours postsurgery. Meperidine was injected at a dose of 0.25 mg/kg with a maximum dose of 0.5 mg/kg if the pain score was higher than 4. Time of the first analgesic request and the total analgesic dose were recorded for each patient. In case of nausea and vomiting, metoclopramide was administered intravenously. The number of recurrences of nausea and vomiting, abnormal bleeding from surgery site, or any complications was recorded for each patient. Patients were excluded if they required secondary surgery or had abnormal bleeding.

### 2.6. Randomization Method

Quadratic block randomization method was used. For this purpose, the researcher provided four envelopes of sheet. On the two sheets, the letter I means “Intervention,” and on the other two sheets, the letter C means “Comparison” was recorded. The envelopes were mixed together and placed in a drawer. The research assistant without any knowledge about the envelopes referred to each eligible patient, and one sheet randomly was drawn, and based on this sheet, whether I or C, patients were assigned to one of the intervention or control groups. It should be noted that the envelopes that drawn out were not returned to the drawer until all the envelopes drawn out. After randomly pulling out all four envelopes, all the envelopes are returned to the drawer again and the procedure was continued again for the next four patients until the desired sample size was reached.

### 2.7. Blinding Method

An independent researcher (nurse) makes random allocation cards (I and C). The sheets were putted in the envelopes and mixed together and placed in a drawer. Another independent researcher referred to each eligible patient, and one envelope randomly was drawn, and based on this sheet, whether I or C, patients were assigned to one of the intervention or control groups. The patients were not aware of the envelope detail. Medications were provided by an independent technician and putted into envelopes based on the allocation orders. Another independent researcher opened the envelopes and informed the doctor about which drug should be prescribed for the patient. In both intervention and control groups, intravenous acetaminophen was prescribed 2 minutes before surgery, so the patient was not informed of the prescription drug. In addition, the assessor that measured the outcomes was not aware of the group's allocation. Therefore, the study was conducted in a triple-blind manner.

### 2.8. Statistical Analysis

Data collected from questionnaires was entered into the statistical package for social sciences (SPSS) software version 21. Descriptive analysis was performed using tables, graphs, ratio, and percentage. Comparison of the nausea, vomiting, and analgesic usage was performed using the chi-square or Fisher exact test. The mean of pain score was compared between study groups using nonparametric test, the Mann-Whitney test. The mean time to first analgesic use and the number of analgesics used by each patient were compared between study groups using the independent sample *t*-test. Normality of data was assessed using the Kolmogorov-Smirnov test. The confidence limit was considered as 95%, and the statistical significance was set as *p* < 0.05.

## 3. Results

A total of 53 patients participated in the acetaminophen and ondansetron I (*n* = 27) and intravenous acetaminophen C (*n* = 26) groups. Demographic characteristics of study patients are presented in Table 1. There was no significant difference between groups in terms of gender, age, weight, and surgery duration.

The frequency of nausea and vomiting in the recovery unit was significantly higher in the intervention group compared to the control group (*p* = 0.010) (Table 2). The Fisher exact test revealed no significant between group differences in frequency of vomiting at recovery unit and after 6, 12, and 24 hours after surgery (Table 3).

**Figure 1:**
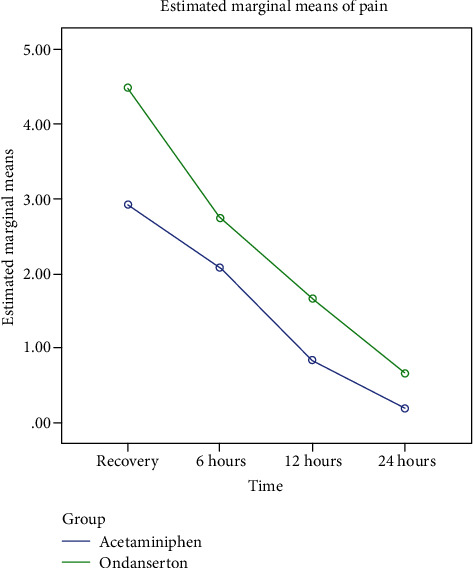
Pain severity score (VAS) in different times after surgery in two study groups.

The nonparametric Mann-Whitney test revealed that the mean pain severity score was significantly different between groups at recovery unit and after 6, 12, and 24 hours after surgery (*p* < 0.05) (Table 4). Repeated measure ANOVA was tested to show pain severity in different times of the study (recovery, 6, 12, and 24 hours after surgery) between two groups. According to the results of this test, both of the factors (time and time^∗^ group) had significant effects (*F*: 4.12, PV < 0.001 for time factor and *F*: 5.38, PV = 0.02 for time^∗^ group factor) (Figure 1).

Table 5 shows that there was no significant difference between groups in terms of the need for analgesics and time to first analgesic request. The frequency of analgesic use by each patient was significantly higher in the intervention groups compared to the control group (*p* = 0.027) (Table 5). Regarding complications, two groups were similar and one patient in each group experienced agitation in recovery room.

## 4. Discussion

This study is aimed at assessing the effect of ondansetron on the analgesic action of intravenous acetaminophen after tonsillectomy in children.

This study revealed no significant difference between intervention and control groups in terms of demographic characteristics (age, gender, height, etc.). In this study, subjects were in a similar status in terms of demographic variables in order to reduce bias in the results. This finding was in line with the findings of the study by Ramirez et al. [6].

Surprisingly, the frequency of nausea in the recovery unit was significantly higher in the intervention group who received ondansetron, an antiemetic drug plus acetaminophen compared to the control group but this difference was not significant in later assessments. There was no significant difference between groups in terms of vomiting. These findings were to some extent similar to the findings of the study by Aydin et al. [13]. This might be due to the medication half-life, which results in the maintenance of the drug plasma concentrations for some time after surgery, and another reason might be the higher pain score in the intervention group.

The findings of the current study revealed that the pain severity score and analgesic dose were significantly higher in the intervention groups compared to the control group at recovery unit and 6, 12, and 24 hours after surgery. A randomized controlled trial by Koyuncu et al. on patients undergoing hysterectomy reported higher pain severity score in the ondansetron group. The authors reported that the analgesic effects of acetaminophen are due to the effect on serotonergic receptors, and that ondansetron significantly reduces the analgesic effects of acetaminophen in the primary stages of postoperative period. The antagonistic effects of ondansetron on acetaminophen were due to the short half-life of the drug [12]. Although the type of surgery was different in the current study and the study by Koyuncu et al., the findings of the studies, including the reduced analgesic effects of acetaminophen and increased need for analgesics, were similar.

In the clinical trial performed by Jokela et al. in Finland, the effect of ondansetron on the analgesic action of acetaminophen was assessed. The study reported that the pain severity score in the ondansetron group was similar to the placebo group, and that ondansetron did not affect the analgesic effects of acetaminophen [14]. The target population of the study by Jokela et al. was hysterectomy patients. This finding was in contrast to the findings of the current study. The reason for this difference might be due to the age range of the subjects as well as differences in the gender, type of surgery, and ondansetron dosage.

In the study by De Witte et al., the mean administered dose of tramadol was significantly higher in the ondansetron group compared to the group who did not receive ondansetron [15]. Similarly, in the current study, the mean dose of analgesic was significantly higher in the intervention group compared to the control group.

In the clinical trial by Ramirez et al., the interaction between ondansetron and acetaminophen after tonsillectomy was assessed. They reported an antagonistic interaction between setrons that are 5-HT3 antagonists and have antiemetic and acetaminophen that stimulate the serotonergic pathway. They reported a significantly higher morphine use based on the PACU pain score in the ondansetron group compared to the group who only received acetaminophen. They mentioned that the analgesic effects of acetaminophen are reduced by the coadministration of setrons [6]. Similar to the findings of the study by Ramirez et al., our study of the effect of acetaminophen and normal saline was compared to acetaminophen and ondansetron in children undergoing tonsillectomy and yielded similar findings. In our study, the mean analgesic dose was higher in the intervention group compared to the control group but this difference was not statistically significant.

The mechanism of action for acetaminophen is not yet fully understood. Few systems are hypothesized for the mechanism of action for acetaminophen including serotonergic, cyclooxygenase, opioid, and cannabinoid systems. Acetaminophen produces its analgesic effects through various pathways including inhibition of prostaglandin E2, COX related, indirect activation of cannabinoid CB1 receptors, and inhibition of nitric oxide system through N-methyl d-aspartate and substance p. The analgesic effects of acetaminophen might be primarily due to the stimulation of descending serotonergic pathways that inhibit signal transduction in the spine [12]. Regarding the acetaminophen-ondansetron interaction, ondansetron does not reduce the plasma concentration of acetaminophen; therefore, the interaction is more due to pharmacodynamic effects compared to pharmacokinetic. Evidence indicates that the serotonergic part of the analgesic effect of acetaminophen is stronger but 5-HT3 antagonists significantly affect the potency of acetaminophen, and that ondansetron even in less potent doses also reduce the analgesic effects of acetaminophen [16].

## 5. Conclusion

Overall, the findings of the current study revealed that intravenous administration of ondansetron at the dose of 0.1 mg/kg in children reduces the analgesic effects of acetaminophen. Furthermore, ondansetron increases the complications including nausea in the first hours after surgery and requirements of rescue analgesics. Therefore, coadministration of ondansetron and acetaminophen is not recommended, and it is better to use other antiemetic medications. It is recommended for further researchers to conduct multicenter studies with a larger sample size on various age groups to assess the mechanism of action of ondansetron on intravenous acetaminophen.

## Figures and Tables

**Table 1:** Demographic characteristics of study patients.

Variable	Intervention (mean ± SD)	Control (mean ± SD)	*p*
Female, *n* (%)	8 (30.8)	11 (40.7)	0.569^∗^
Age (years)	7.17 ± 3.04	7.04 ± 2.56	0.767^∗∗^
Weight (kg)	22.29 ± 7.49	23.30 ± 9.56	0.660^∗∗^
Height (cm)	111.46 ± 18.79	118.48 ± 26.12	0.268^∗∗^
Surgery duration hours (minutes)	01.17 ± 00.25	01.03 ± 00.20	0.056^∗∗^

^∗^Chi square. ^∗∗^Student's *t*-test. ^∗∗∗^Sex variable defined as percent.

**Table 2:** Frequency distribution of nausea in recovery unit till 24 hours after surgery in intervention and control groups.

Time (minutes)	Intervention n (%)	Control *n* (%)	*p*
Recovery	6 (23.1)	0 (0)	0.010^∗^
6 hours	3 (11.1)	7 (26.9)	0.175^∗^
12 hours	1 (3.7)	1 (3.8)	1.00^∗^
24 hours	0 (0)	0 (0)	1.00^∗^
Total	4 (18.4)	9 (24.6)	0.094^∗∗^

^∗^Fisher exact test. ^∗∗^Chi square.

**Table 3:** Frequency distribution of vomiting at recovery till 24 hours after surgery in intervention and control groups.

Time (minutes)	Intervention *n* (%)	Control *n* (%)	*p*
Recovery	0 (0)	1 (3.8)	0.491^∗^
6 hours	1 (3.7)	3 (11.5)	0.351^∗^
12 hours	1 (3.7)	1 (3.8)	1.00^∗^
24 hours	0 (0)	0 (0)	1.00^∗^
Total	2 (7)	5 (19.2)	0.250^∗^

^∗^Fisher exact test.

**Table 4:** Comparison of the pain severity score between groups at recovery, 6, 12, and 24 hours after surgery.

Pain severity	(Mean ± SD)		Median (Q1-Q3)		*p*
Time	Intervention	Control	Intervention	Control	
Recovery	4.48 ± 2.04	2.88 ± 1.07	4 (3-6)	3 (2-3)	0.001^∗^
6 hours	2.74 ± 1.26	2.04 ± 1.11	3 (2-3)	2 (1-2)	0.012^∗^
12 hours	1.67 ± 0.88	0.81 ± 0.75	2 (1-2)	1 (0-1)	<0.001^∗^
24 hours	0.67 ± 0.55	0.020 ± 0.50	1 (0-1)	0 (0-0)	0.001^∗^

^∗^Mann-Whitney test.

**Table 5:** Frequency distribution of the analgesic usage and first time analgesic administration in the intervention and control groups.

Variable	Intervention	Control	95% CI	*p*
Frequency of analgesic requirement *N* (%)	19 (70.4%)	13 (50.0%)	------	0.120^∗^
Time to first analgesic need (min) (mean ± SD)	157 ± 166	130 ± 132	-75.5-29.7	0.594^∗∗^
Number of analgesic intake by each patient	1.07 ± 0.94	0.56 ± 0.30	0.06-0.94	0.027^∗∗^

^∗^Chi-square test. ^∗∗^Student's *t*-test.

## Data Availability

All data generated or analyzed during this study are included in this published article.
